# Nudging Strategies for Arable Land Protection Behavior in China

**DOI:** 10.3390/ijerph191912609

**Published:** 2022-10-02

**Authors:** Yanwei Zhang, Xinhai Lu, Yucheng Zou, Tiangui Lv

**Affiliations:** 1 College of Public Administration, Huazhong University of Science and Technology, Wuhan 430074, China; 2 College of Public Administration, Central China Normal University, Wuhan 430079, China; 3 School of Public Affairs, Zhejiang University, Hangzhou 310058, China; 4 School of Tourism and Urban Management, Jiangxi University of Finance and Economics, Nanchang 330013, China

**Keywords:** arable land protection behavior, nudging strategies, behavioral intervention, theoretical discussion, China

## Abstract

Arable land protection is critical to the sustainable development of agriculture in China and acceleration of the realization of the trinity protection goal of the quantity, quality, and ecology of arable land. As a new program of behavioral science to promote social development, nudge has gradually gained the favor of researchers and policy makers due to its unique advantages of small cost and substantial effect. However, current research and practical exploration of arable land protection behavior intervention based on the idea of nudging are still lacking. Implicit nudging strategies directly target the heuristic and analytic systems of arable land protection behavior of each stakeholder and possess more advantages than traditional intervention strategies. Therefore, this article designs six arable land protection behavior nudging strategies from the perspectives of cognition and motivation to realize the theoretical discussion of “generating medium-scale returns with nano-level investment”. The nudging strategies of the cognitive perspective include default options, framing effects, and descriptive norms, while those of the motivation perspective aim to stimulate home and country, and heritage and benefit motives to promote arable land protection behavior of various stakeholders. The utility of nudge to arable land protection behavior may be controversial in practice. Therefore, the implementation in China should be based on the division of farmers, the number of options should be appropriate, and the external environment of arable land protection behavior should be fully considered.

## 1. Introduction

Arable land is not only the most powerful guarantee for national food security but also determines the coordination and sustainability of socio-economic development and ecological environmental protection in a country or region to a large extent [1]. Food security is a national strategy in China, and arable land protection is especially important for such a country with a population of 1.4 billion [2,3]. Therefore, the No. 1 Document of the Central Committee of the Communist Party of China was proposed in 2019 to stabilize grain production, fully implement the special protection system for permanent basic farmland, and ensure the establishment of 800 million mu (mu, Chinese measurement that is commonly 666.7 square meters) of high-standard farmland by 2020. However, the contradiction between limited arable land resources and the construction land expansion has become increasingly serious with the acceleration of industrialization and urbanization, and the massive loss phenomenon of arable land resources has intensified [4,5]. Simultaneously, improper use of arable land and environmental pollution have degraded arable land quality, thus also becoming increasingly serious [6,7]. The survey shows that the degraded area of arable land accounts for more than 40% of the total arable land area at this stage, the productivity of arable land has declined, and the over-standard rate of soil heavy metals has reached 1.4% (data from the Ministry of Agriculture and Rural Affairs of China in 2018). Arable land users are the key stakeholders of the quantity, quality, and ecological investment of arable land [8,9]. A gap still exists between arable land protection behaviors and policy expectations despite the improvement of the micro-level arable land protection practices under the impetus of laws and regulations, No. 1 Central Documents, and various special plans [10,11]. Therefore, encouraging micro-stakeholders to adopt arable land protection measures actively is necessary to restore and improve the health level of arable land in China.

The central government is the maker of arable land protection policies and the ultimate regulator of arable land protection activities [12]. The central government focuses on long-term sustainability and stock in the management of arable land, hoping that different stakeholders (local governments and farmers) can use arable land in a balanced manner over time. Facing the strategic behavior of illegal occupation of arable land by local governments or farmers, the central government uses command-control and economic incentive tools to restrain and encourage the spontaneous arable land protection behaviors of various stakeholders [13,14]. Command-control tools are backed by national compulsory power [15] and directly stipulate the production behavior and utilization methods of arable land users through administrative orders or established regulations and standards. The economic incentive tool aims to use economic means or market forces to take subsidy measures or establish price mechanisms for arable land protection behaviors (such as soil fertilization behavior of farmers and transformation behavior of weak arable land) to realize the internalization of the negative externalities of arable land use [16]. These strategies for protecting arable land can be attributed to the two paths of carrot and stick, which belong to traditional social governance methods [17]. However, insurmountable difficulties are found in the design and implementation of simple paternalism management methods due to the differences in the interests of the main stakeholders of arable land protection. Therefore, a new low-cost and non-mandatory incentive strategy for arable land protection behavior should be formulated.

The essence of arable land protection is a management activity involving multiple subjects. However, due to the problems of inconsistent goals, non-equilibrium incentives, and differences in constraint pressures among various types of subjects in the process of arable land protection, it is easy for different subjects to take actions for their own interests in the process of arable land protection. The bad result is to gradually distort the original intention of arable land protection goal setting, and ultimately lead to the failure of the arable land protection policy. Therefore, the protection of arable land is inseparable from the joint efforts of multiple subjects. It is necessary for everyone to work together around the common goal of arable land protection, cooperate with each other, and finally form a joint force. Moreover, from the perspective of governance, how to promote the interaction of the participation of various subjects and mobilize the endogenous power of different subjects has become an important direction and path for the arable land protection in the future [18]. The main subjects of arable land protection include the central government, local governments, and farmers. The interest of central government is to ensure food security. In order to stimulate the endogenous motivation of local governments to protect arable land, while restraining their opportunistic behaviors, the central government implements a two-pronged management model of strict supervision and enhanced compensation to ensure the occurrence of arable land protection behaviors. The interests of local governments are the economic development and promotion trophies. Under the triple pressure of food security, economic performance and political promotion, local governments usually do not incorporate food security into the objective function, but are more inclined to the huge benefits brought by land finance. Therefore, local governments will adopt flexibility and collusion to deal with the goal of arable land protection, resulting in the dilemma of arable land management. The interests of farmers are to maximize their own interests. The comparative benefits of farmers operating arable land are low and the expected returns are unstable, which makes farmers gradually separated from the agricultural sector to the non-agricultural sector driven by economic interests, resulting in a serious shortage of labor force and a serious aging phenomenon in rural areas. Despite land attachment or limited non-agricultural skills, there are still some farmers who continue to engage in agricultural production by transferring, taking over, or renting the arable land of the remaining farmers. However, in view of the harsh natural environment, the serious marginalization of arable land, and the psychological gap brought about by low agricultural returns, agriculture has weakened and arable land has become “non-food”, “non-agricultural”, “abandoned” and other phenomena. Therefore, arable land protection is essentially a behavioral planning issue of stakeholders from the perspective of behavioral science, and behavior is the result of individual decision-making choices [19]. Influencing individuals to make correct decisions is an effective way to intervene individual arable land protection behaviors to increase the scale of arable land, improve arable land quality, and enhance the arable land structure. Behavioral economists Richard H. Thaler and Cass R. Sunstein first proposed the concept of nudge in 2008; that is, a nudge is any aspect of the choice architecture that alters people’s behavior in a predictable way without forbidding any options or significantly changing their economic incentives [20]. The boost strategy integrates psychology and behavioral economics into public policy making, avoiding the shortcomings of pure paternalism or libertarian. This strategy is neither carrot nor stick; thus, it is called the fifth way of social governance (the four remaining roads are hierarchy, markets, networks, and persuasion) [21]. The multiple advantages of the nudging strategies have received extensive attention from the academic community and the government. At present, applied research has been conducted in various fields, such as health (including a healthy diet, medical treatment and health, and weight loss), environmental protection, social security, education, and charity, and has shown good application value [22,23,24,25]. Nudging is also applicable in the field of arable land protection behavior. For example, the central government can establish an assessment mechanism linking arable land protection goals with political performance, thereby enhancing the role of arable land protection in the performance assessment of local governments and mobilizing the inherent incentives for arable land protection. In addition, the central government can also strengthen dissemination on the effectiveness of arable land protection through publicity and guidance tools, so that more farmers can truly recognize and understand the market value and non-market value brought by arable land protection. The publicity and guidance tools are to disseminate the arable land protection policy by means of media, so as to reduce the cost of arable land protection, reduce the pressure of policy implementation, strengthen the consensus of various subjects on the applicability of the policy, and then consolidate the behavior of farmers to participate in arable land protection. Effective policy dissemination can not only reduce farmers’ indifference or resistance to arable land protection, but also enable the government to obtain timely feedback from public opinion to improve the policy content. There are still many typical nudging tools. In reality, different nudging strategies should be adopted according to different arable land resource endowments and different social scenarios, and in many situations, it is even a comprehensive application of many nudging strategies. Thus, this article aims to provide a simple and low-cost architecture choice through nudge. Therefore, arable land protection behavior will change in the expected direction, and an attempt to generate medium-scale returns with nano-level investment can be realized.

## 2. Nudge Theory

### 2.1. What Is Nudge?

Behavioral economics has three basic assumptions concerning human nature: limited rationality, willpower, and self-interest. The decision-making rationality of humans is limited, not only restricted by the limitation of knowledge but also by the decision-making environment. The limitations of human cognitive abilities, such as greed, impulsivity, inertia, and other weaknesses, lead to various cognitive biases, such as selective perception, proximity effect, correlation fallacy, and overconfidence in the judgment of decision-making [26]. The decision-making choices demonstrate abnormal phenomena, such as loss aversion, contention with the status quo, and short-term preferences. In addition, human behavior is often affected by social factors and cannot be truly autonomous. Thus, people often encounter difficulties when facing complex and major decision-making issues, failing to make correct decisions that conform to their wishes and well-being.

Therefore, how should government managers deal with the systematic biases of human behavior? The paternalism management method advocates that individuals lack rationality and self-control and supports mandatory restraints on individual behavior [27]. Meanwhile, the liberalism management method advocates that the individual’s right to choose is inalienable and does not approve of mandatory intervention in individual behavior [28]. The nudge management method advocates the concept of libertarian paternalism, which finds the factors that are ignored by traditional economists in the selection environment to influence individual decision-making choices while ensuring that free choice of individual decision makers is not reduced, and objective payment and remuneration remain unchanged. Thus, individual decision-making will develop in the direction of improving personal and social welfare. [29]. The typical nudge is the change in choice architecture. The core of choice architecture is that policy makers create specific situations and change specific conditions by grasping the psychology of policy executors; thus, the latter can make decisions according to the hope of the former. Moreover, this process is low cost and highly rewarding, which is similar to “nudging with an elbow or other parts of the body”, making it easy for people to do what they want [30].

Nudge attempts to influence and change the decision-making behavior of the public with small non-mandatory measures by understanding the psychological mechanism of behavior. Thus, on the basis of maximizing resource saving, nudge plays an important role in reducing impulsive behavior and improving rational behavior. Intervention on individual behavior through the nudge method reveals the small entry point but grasps the nature of the problem from the behavior, and the behavior stems from the choice. Therefore, the rational use of nudging strategies can promote social development.

### 2.2. Why Do We Need Nudge for Arable Land Protection Behavior?

Currently, the nudging theory has been widely used by different scholars in different fields around the world. For example, Zhang et al. [31] argue that effective diffusion of electric vehicles could help China achieve carbon neutrality by 2060. Moreover, the paper points out that the combination of nudging policies and charging infrastructure can have a greater publicity effect than subsidies for car purchases. In other words, the role of nudging policies in information promotion helps Chinese people to accept electric vehicles more easily. Wang [32] believed that humans are often unable to make optimal behavioral decisions due to their limited attention span and limited computing power. With the development of behavioral economics, nudge has become an important tool to improve human irrational behavior and ultimately achieve happiness. Among them, commitment devices and default options can help people stick to their decisions; social comparison and incentives can encourage people to realize their behavioral intentions; message framing and simplifying complex information can lead to increased service usage. The flexible use of nudging strategies by social workers can facilitate policy formulation and practice design. Chen et al. [33] proposed that ozone pollution poses serious health risks and premature death. Additionally, gas stations are a significant source of organic compounds released by cities. The government’s call on car owners to refuel at night is one of the important strategies for green nudging. The results of its research show that the preferential policy of refueling at night will contribute to the reduction of ozone concentration and bring great benefits to human health. It can be seen that the nudging strategies consider both the motivation and control of human behavior, and can effectively change people’s behavior through the ingenious design of some mechanisms. In turn, it promotes people to make certain behaviors that meet specific goals, but at the same time does not compromise people’s freedom of choice. Moreover, the core of nudging is to influence and change the behavioral decision-making of the public with non-coercive measures by understanding the mechanism of psychology. On the basis of saving resources to the greatest extent, it can help reduce impulsiveness and improve rational behavior [34,35,36]. Therefore, this paper tries to propose nudging strategies based on behavioral economics by analyzing the status quo of arable land protection behaviors of various subjects in China.

Different from daily life decision-making, people have relatively little knowledge in the field of arable land protection. For example, people mistakenly believe that soil pollution can quickly disappear with the reduction in the large-scale use of fertilizers and pesticides. However, unlike mobile pollution, such as water and gas, soil environmental degradation, pollution, and hazards have the characteristics of accumulation, concealment, and inhomogeneity. Unless it is removed and repaired by manual measures, it will remain for a long time and cause various hazards along with the arable land use [37,38]. In addition, people generally blindly believe that the arable land ecosystem can be restored through governance measures. However, the destruction of arable land due to natural disasters, arable land abandonment, and extensive use of arable land has become an irreversible process of land degradation [39,40].

**Figure 1:**
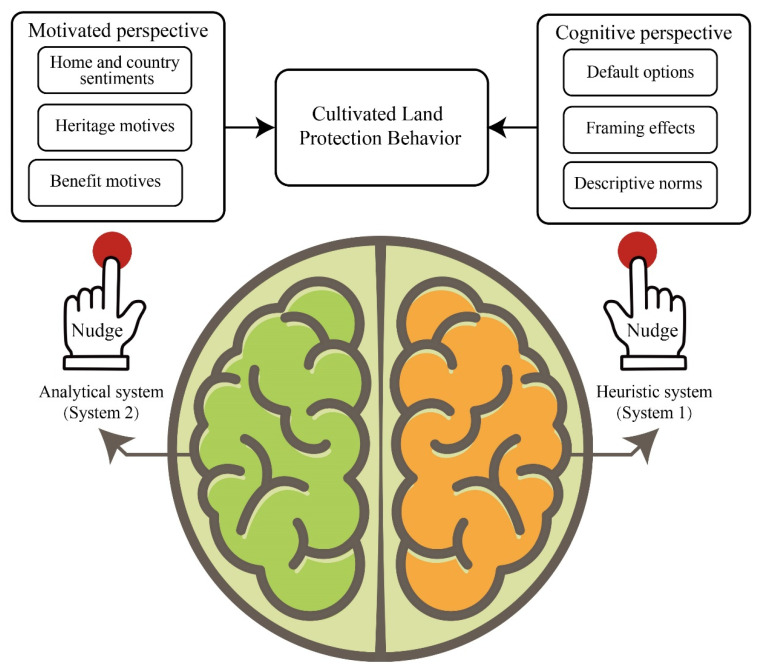
Nudging Strategies for Arable Land Protection Behavior in China.

Psychologists believe that human judgment and decision-making usually involve two major cognitive systems: a heuristic system based on intuition (system 1) and an analytical system based on reason (system 2) [41] (Figure 1). System 2 is characterized by its consciousness, energy consumption, and control. This system needs to mobilize attention to analyze and solve problems. Moreover, this system is not prone to errors despite its slow operation [42]. The formulation of traditional intervention strategies for arable land protection is mostly based on the assumption that individuals are rational people, that is, individuals are believed to be able to use system 2 to conduct rational analysis and take the behavior of sustainable use of arable land [43]. However, numerous studies in behavioral economics show that the process of individual judgment and decision-making is not completely rational [44]. Especially in the case of relatively limited knowledge in the field of arable land protection, using the information processing mode of system 2 to make decisions becomes fairly difficult, and people are inclined to implement rapid automated decision-making based on system 1. Compared with system 2, system 1 is a conscious and automated system, which runs fast and is full of emotions. However, people often focus on the short-term benefits of arable land use and neglect long-term considerations, leading to phenomena, such as arable land pollution, arable land desertification, and soil erosion [45]. Moreover, people have substantially limited experience and are prone to decision-making errors due to the deterioration of the arable land ecological environment and the long and complex dynamic change process of arable land spatial patterns [46]. Thus, nudge is a necessary strategy for decision-making and behavioral intervention.

In addition, the result of the trade-off between the input cost and the expected benefits of arable land protection determines the behavioral decisions of various stakeholders despite the sufficient knowledge of people in the field of arable land protection. Arable land protection costs mainly include direct input, non-agricultural opportunity, and policy implementation costs; the benefit is to guarantee the space for the future social economy and sustainable development of the region [47]. The behavioral cost of arable land protection is currently certain, but its benefits will be full of uncertainty in the future. This asymmetry between costs and benefits easily lowers the motivation of people to protect arable land [48]. In addition, this asymmetry is reflected in that the cost belongs to the stakeholders of arable land protection, while the benefit belongs to the society. As a special ecosystem, arable land has the attributes of public goods. The ecological (such as water conservation, soil and water conservation, climate adjustment, and environmental purification) and social (such as ensuring food security and maintaining social stability) benefits produced by arable land have not been included in the benefits of arable land use due to the characteristics of universal supply, non-exclusion, and non-competitiveness of public goods. This imperfect arable land use mechanism reduces the enthusiasm of stakeholders to protect arable land, which makes it impossible to realize the non-market value of arable lands, and contributes to the lack of public welfare existing in the externality of arable land [49,50]. A variety of specific measures can be formulated for the objectives of arable land protection based on the flexibility of nudging. These measures can circumvent the lack of motivation of the stakeholders of arable land protection and promote their effective decision-making.

People have relatively minimal knowledge and experience in the field of arable land protection, thus relying on system 1 for decision-making. However, the decision path that relies on system 1 has the characteristics of modular closed operation, automatic response, and susceptibility to stereotyped impression, and this path is prone to unreasonable behavior. By contrast, the costs and benefits of arable land protection are asymmetrical at the two levels of present–future and internality–externality, which lead to insufficient micro-motivation and enthusiasm of relevant stakeholders of arable land protection. Therefore, arable land protection behavior can be nudged from the following four perspectives. (1) Cognition is the prerequisite for various stakeholders to participate in the arable land protection. The improvement of cognition level plays a decisive role in its willingness to pay for participating in arable land protection. The increase in the various values of arable land can introduce benefits and welfare improvements to the stakeholders of arable land protection. Therefore, the psychological source of unreasonable arable land protection behaviors is emphasized and nudging measures are used to avoid cognitive biases and abnormal choices of decision makers, thus achieving the purpose of changing behaviors of stakeholders. (2) The policy design of arable land protection should conform to the psychological laws of decision-making of various stakeholders. Moreover, the choice architecture should be reasonably designed to guide the stakeholders to change their arable land protection behaviors to conform to individual interests and social well-being. (3) From the perspective of psychological cognition, the individual’s perception of arable land protection behavior is accompanied by the cognitive process comprising elements, such as feeling, perception, memory, thinking, and imagination. Introducing the analytical framework of behavioral economics into the field of arable land protection behavior has theoretical consistency and necessity. (4) “Very cherish, rational use, and effective protection of arable land” has become the basic national policy in China, and improving the quality and efficiency of arable land use has become an important theme of the policy formulation of the Chinese government. However, behavior-based intervention mechanisms in the existing arable land protection policy toolbox are still lacking. Therefore, one of the most appropriate reasons for introducing the nudging mechanism in the field of arable land protection behavior is to incorporate the psychology, motivation, and cognition of each stakeholder into the policy action framework and establish arable land protection as a solid foundation for the continued prosperity of rural areas and the happy lives of farmers.

In addition, the current restraint mechanism of arable land protection mainly relies on the top-down management mode. The reason is that the conventional public governance model usually fails when intractable public crises and public problems arise. At that time, it is necessary to force the decree through the administrative force, and then decompose and manage the pressure-oriented goals and tasks from the top to the bottom in the bureaucratic structure. As a public resource, arable land has the general attributes of a public resource. Taking a top-down approach in the management process can effectively reduce ambiguity and randomness, thereby ensuring the implementation of policy goals. However, in the top-down arable land protection management mode, there are two obvious defects. First, for a bureaucratic organization with complex and super-large scales, long information dissemination channels and multiple principal-agent mechanisms responsible for each level can easily lead to the absence of arable land protection supervision. Second, this approach to arable land protection pays too much attention to the compulsory control of the government, and tends to ignore the initiatives, demands and actual conditions of other subjects. This easily leads to the diversity, deviation and uncertainty of the actual action results, which has been widely criticized by the academic circles. As a bottom-up management method, nudging tends to pay more attention to the arable land protection process of each subject at the micro-scale. The most important feature of nudging is: nudging can pay attention to the practical problem of the inconsistency between the overall system design of arable land protection and the micro-behavior level. Arable land protection can produce windfall gains or wipe-out losses, resulting in uneven interests of land users. Agricultural production on arable land is not only inefficient in terms of economic benefits, but its non-market value has certain attributes of public goods. To a certain extent, the protection of arable land sacrifices the opportunities and space for local governments and farmers to develop non-agricultural construction, and gives up the greatest opportunity cost that can be obtained by converting arable land into construction land. Therefore, although traditional administrative intervention strategies can improve the target population’s awareness of the risk of arable land destruction or their willingness to protect arable land in a short period of time, they may not actually lead to effective behavior changes. Moreover, even if the arable land protection behavior can be effectively improved, the time and economic costs required for administrative intervention, economic intervention and continuous monitoring are huge. Therefore, perhaps we can learn from the nudge theory to carry out empirical, unconscious, and automatically triggerable arable land protection behaviors, and then turn them into habits to help people overcome the gap between arable land protection intentions and arable land protection behaviors. Drawing on the toolkits used in past nudging, this study divides nudging mechanisms into six categories: default option, framing effects, descriptive norms, home and country sentiments, heritage motives, and benefit motives. The default option refers to re-examining the existing default options and taking arable land protection as a default option with potential economic, social, and ecological benefits, so as to improve the possibility of each subject taking arable land protection behaviors. The framing effect refers to the phenomenon that different representations of the same information lead to different decision-making effects. The framing effect in arable land protection refers to the phenomenon that the decision-making behavior of each subject is affected by the media or leaders’ frame representation of cultivated land protection issues, and shows different decision-making preferences. The descriptive norm refers to the obvious role model effect and group effect among various subjects in the process of arable land protection. It reflects that the attitude and participation enthusiasm of a subject towards arable land protection will have a significant impact on whether other subjects continue to participate in arable land protection. For example, inter-neighborhood exchanges and demonstrations can increase farmers’ willingness to apply environmentally friendly technologies more than government policy interventions. The home and country sentiment refers to the moral rationality that emphasizes the value and meaning of individual life must rely on the value and meaning of the country, and also refers to the individual’s psychological, emotional attachment and satisfaction to family, hometown, and patriotic feelings. The Chinese people have a strong sense of home and country, as well as patriotic cultural values and corresponding behavior patterns. Moreover, the arable land protection is also a major support for China’s national security strategy. Therefore, there is a high social consensus on the arable land protection and the guarantee of food security. The heritage motive refers to the economic behavior of older generations to pass on a portion of their income and wealth to the next generation. Arable land has important social security value and social stability value. The elderly farmers pass the arable land to the next generation, in fact, they hope that the income of young farmers will be more diversified. For them, arable land is the last guarantee for the survival of family members and an asset that may greatly appreciate in the future. Benefit motives means that each subject is an independent operating subject pursuing the maximization of their own profits. In view of this, only when each subject believes that arable land protection is profitable, will the supply behavior of arable land protection be increased.

Therefore, nudges can influence people’s choices, but they don’t force people to change their choices, nor do they make choices for people, but help people make better choices at insignificant increased costs. However, due to the constraints of information acquisition, cognitive ability, and self-control, people’s daily decision-making usually shows the characteristics of bounded rationality. People often rely on empirical judgments of various heuristics, and thus often make inefficient decisions that are inconsistent with their own well-being. The nudging strategies is aimed at improving this situation. It is unique in that it does not need to resort to executive orders or economic leverage, but to change people’s behavior in the desired direction by providing an appropriate choice framework. Of course, nudging cannot solve all the problems arising in the process of arable land protection. In practice, it still needs to be managed through tough measures such as arable land occupation tax, arable land dynamic monitoring, and arable land use control. However, nudge is a new insight from behavioral economists based on psychology, and provides a new perspective for understanding and predicting economic behavior. The purpose of this study is to try to use nudge (this low-cost and high-efficiency regulatory method) to intervene in the micro-level arable land protection behavior more finely, so that each subject can make decisions in a more optimized way, thereby improving the arable land environment.

## 3. Cognitive Perspective of the Nudging Strategies of Arable Land Protection Behavior

The cognitive perspective of the nudging strategies of arable land protection behavior aims to avoid the cognitive bias and abnormal selection of decision makers by designing a reasonable choice architecture to promote their rational arable land protection behaviors. This article focuses on the application of default options, framing effects, and descriptive norms in the field of arable land protection behavior. Default options and framing effects can encourage the arable land protection behavior by cleverly presenting decision-making information, while the descriptive norms promote arable land protection behavior through customized information.

### 3.1. Default Options Nudge Arable Land Protection Behavior

The default option refers to the option to be accepted when the individual has not yet made a decision [51]. The decision will be affected by the framework in the absence of a formed value or preference of an individual, and the default option is then used as a reference point [52,53]. Therefore, people tend to keep the default options without making any changes during decision-making. This phenomenon is the default option effect. In the application and research of public policies, the nudging strategies of default options are widely used in environmental protection [54], consumer food choices (Just et al., 2018), and public health [55]. Policy designers should focus on the use of default options to make minor adjustments in the design of arable land protection policy to cause significant changes in the arable land protection behavior of each stakeholder and then achieve the goal of arable land protection. For example, land-use change caused by the increase in various types of construction land is one of the most significant features of urbanization. The essence of urbanization is the transformation process of land use function. Population agglomeration, industrial structure agglomeration, and infrastructure construction must be realized through the reconfiguration of land [56,57]. However, urban expansion invaded and occupied a large amount of arable and ecological lands, which directly led to a sharp decline in the amount of arable land and the occurrence of ecological and environmental problems, posing a serious threat to food security and ecological protection in China [58]. Therefore, optimizing the allocation of limited land resources and realizing the coordinated development of urban expansion, arable land protection, and ecological conservation is a serious challenge facing sustainable land use in China.

At present, delimiting urban growth boundaries, establishing arable land occupation tax, restricting basic arable land zoning, and setting up land regulatory agencies have become important means to control urban expansion and protect arable land. The central government has paid huge administrative costs and financial investment, and has been improving the governance efforts of illegal activities on arable land yearly. However, illegal cases of arable land became increasingly concealed and challenging to investigate when local officials conspired to participate in such illegal use. In response to this problem, Wu [59] believes that high-, medium-, and low-gradient quota systems can be set in the construction land quota application system according to the development of each region and resource endowments. In the high-gradient quota application system, local governments will face problems, such as a large number of application materials, complicated approval procedures, and long waiting times for approval. The procedure is simplified and the application is easy in the medium- and low-gradient quota application systems, and this option is set as the default. Local governments have the political task of ensuring regional economic development and fiscal balance and reducing unemployment and social stability. Thus, they may not choose the low-gradient option. However, the local government may choose the default option because the high-gradient option has the characteristics of complexity, rigorous approval process, and prohibitive length of approval time.

Cases in real life, wherein the absolute superiority option (that is, a better option than others in all dimensions) is among the choices that people face, are relatively few. Most decision-making tasks involve the comparison of alternatives with default options and objectively equivalent losses and gains. People tend to regard the default option as a reference point during decision-making. Judgments of people regarding losses and gains are prone to change due to reference point dependence. People argue that the loss of abandoning the default option is larger than the benefit of choosing an alternative option (loss aversion) [60]. The individual subjective susceptibility caused by the loss is substantial; thus, people usually keep the default option and are unwilling to make changes to avoid the psychological loss caused by abandoning the default option, leading to the generation of the default effect (settle for the status quo) [61]. The above-mentioned characteristics of human habitual thinking provide a practical idea for nudging arable land protection behavior. That is, replacing traditional options with arable land protection ones as the default, thereby guiding people to make arable land protection behaviors.

### 3.2. Framing Effects Nudge Arable Land Protection Behavior

The framing effects can also effectively avoid the cognitive bias in decision-making caused by human loss aversion, thereby nudging arable land protection behavior. The framing effects mean that attitudes and preferences of people toward the event will change or even be reversed when presented with essentially the same events only because of the modified way of presentation. That is to say, different ways of expressing the same problem may cause individuals to make different decision-making results. Researchers generally believe that intuitive experience and emotional preference, which are crucial for the decision-making system, are the underlying causes of framing effects [62]. This section mainly discusses the influence of delay–advance and goal framing effects on the behavioral decisions of various stakeholders in the arable land protection.

The delay–advance framing effects indicate that people have different perceptions of waiting time under delayed and advanced conditions due to the reference point. Therefore, delays and advances are often regarded as losses and gains, respectively [63,64]. The agricultural subsidy policy, which aims to protect and develop agriculture, is an important program of strengthening and benefiting farmers in China [65]. At present, adjusting the agricultural subsidy policy and linking the issuance of various agricultural subsidies with the effect of arable land protection, which forms an agricultural subsidy system with arable land protection as the core, plays an important role in increasing the income of farmers and ensuring national food security. However, the issuance time of agricultural subsidies in China significantly varies in different regions. Some regions can be issued before the end of March, while others have to be delayed until the end of April or even later. At present, in the face of continuously increasing agricultural production materials, the government needs to issue agricultural subsidies promptly to ensure the enthusiasm of farmers for arable land protection. Therefore, governments at various levels should adjust the timing of the issuance of agricultural subsidies to the beginning of the year to mobilize the enthusiasm of farmers for growing grain effectively and prevent the increasing phenomenon of non-grainization.

The goal framing effects refer to the changes in the willingness of individuals to implement the behavior when describing the relationship between the implementation or non-implementation of certain behavior and the realization of the goal [66]. For example, the value of arable land mainly includes economic production, social security, ecological conservation, and cultural inheritance [67,68]. Among these values, economic production value can provide farmers with agricultural economic income and agricultural products; social security value can provide farmers with employment opportunities and reduce the risk of farmers going out to work; ecological conservation value can consolidate the foundation of agricultural reproduction and reduce the loss of agricultural output and production costs; and cultural inheritance value can increase the recognition and respect of farming culture by future generations. Therefore, the arable land protection behavior can be described as follows: “if you protect arable land, you will increase family income, increase employment security, reduce reinvestment in agricultural production, and be praised by future generations” or “if you do not protect arable land, then you will significantly reduce the level of agricultural income, lose the most basic social security, increase the cost of agricultural production, and is not conducive to ensuring the livelihood of future generations”. Therefore, the information expression of the goal framing can significantly affect the willingness of farmers to protect arable land.

Different delay–advance and goal framing will generally affect individual arable land protection decisions. In practice, rational use of the framing effects and finding the key variables in these effects can promote the arable land protection behavior of individuals.

### 3.3. Descriptive Norms Nudge Arable Land Protection Behavior

The default options and framing effects nudge the arable land protection behavior of people by masterly presenting decision-making information, while the descriptive norms nudge such behavior by directly providing customized information. When arable land protection behavior becomes a descriptive norm, which is a typical practice of most people in a certain situation, the possibility of individuals taking arable land protection behavior will remarkably increase. Descriptive norms convey information to individuals regarding the behavior of most people in a specific situation. This information is equivalent to telling the individual what to do in a specific situation and is most likely to be effective and suitable, providing a basis for the decision-making of an individual; thus, people can behave in accordance with the behavior of most individuals [69,70].

Conservation tillage refers to a technical system with surface mulch, straw return, and no-tillage as the core technology using comprehensively supporting measures, such as reduced tillage, no-tillage, surface micro-topography modification technology, surface cover, and rational planting [71,72]. Most farmers are subjectively cautious due to their education level and smallholder management restrictions, which is not conducive to the promotion and application of conservation tillage. The producer, who is the first to adopt a new mode of production, faces the largest uncertainty, while the followers encounter a relatively small amount of uncertainty [73]. Therefore, a demonstration is the best way to reduce the uncertainty faced by farmers in adopting conservation tillage. In addition, farmers often make choices based on the adoption of conservation tillage by influential farmers or individuals (such as large-scale growers, cooperative leaders, village officials, and rural elites) in the village. The adoption behavior of this part of farmers has descriptive and silent dissemination effects. Therefore, the government should explore the establishment of a conservation tillage training and promotion mechanism for rural elites while guiding them to play a positive role. Thus, farmers can subtly learn new knowledge of conservation tillage during the communication process to accelerate the adoption and diffusion improvement of new technologies in the social network of farmers.

Therefore, the government should actively build experimental demonstration bases for conservation tillage in various regions and use them as carriers to conduct conservation tillage promotion and new-type professional farmer training and actively cultivate application entities that support conservation tillage. In addition, technology demonstrations can reduce the risk expectations of farmers and increase their enthusiasm for applying conservation tillage technologies.

## 4. Motivated Perspective of the Nudging Strategies of Arable Land Protection Behavior

Two asymmetries are observed in the costs and benefits of arable land protection behavior: “present–future” and “individual–society”. Moreover, under the incentive of the substantial benefits of non-agriculturalization of arable land, each stakeholder lacks the exogenous implementation power of arable land protection behavior due to the imperfection or absence of the incentive mechanism for arable land protection behavior. On the one hand, the home and country sentiments and heritage motives can be stimulated to raise the attention of people to the future food security, the inheritance of farming culture, and the preservation of arable land resources for future generations. Consequently, the “present–future” asymmetry between the costs and benefits of arable land protection behavior can be alleviated. On the other hand, it can stimulate the benefit motives of each stakeholder to enhance their recognition of the multi-functional value of arable land and the arable land development rights. Consequently, the “present–future” asymmetry between the costs and benefits of arable land protection behavior can be alleviated. Therefore, home and country sentiments and heritage and benefit motives can nudge the occurrence of arable land protection behavior.

### 4.1. Home and Country Sentiments Nudge Arable Land Protection Behavior

Adopting arable land protection behavior is the result of weighing the current costs and future benefits of each stakeholder. The stakeholders often act unfavorably to the ecological environment of arable land mainly because they are short-sighted and cannot see the value of sustainable use of arable land. Therefore, they lack the motivation to invest in arable land protection. For example, the behavioral decisions of local governments regarding arable land protection are often inconsistent with their social goals. This inconsistency is mainly due to the inherent requirements of regional economic development goals, which drive local governments to choose to provide land at a low price in the process of attracting investments. Local governments provide excessive attention to the transfer of arable land to achieve the practical needs of fiscal revenue increase [74]. Therefore, a nudging design that allows various stakeholders to see the future value of arable land will encourage their participation in arable land protection behavior. Considering the country, people intuitively perceive a long future for the country when its history is long. This intuitive feeling easily stimulates the sense of responsibility of people for the future of the country [75,76] to allow effective consideration of such future and conduct additional arable land protection behaviors. The feelings of home and country are the quintessence of the traditional culture of the Chinese nation. Awakening home and country sentiments and raising awareness of arable land protection are crucial in the current situation.

The development of rural slogans in rural areas of China is crucial due to the scattered geographical distribution of villages, weak cultural environment, and single access to information. Rural slogans are highly praised by the vast rural areas because of their short, concise, easy-to-remember, easy-to-recognize, and catchy features. Slogans are not only an important and direct teaching material for farmers to learn and understand various policies and guidelines but are also effective in guiding and educating farmers to form correct values. Rural slogans play an irreplaceable role to a large extent [77]. Therefore, the rural slogan can elicit home and country sentiments of people regarding arable land protection (for example, “the land is connected to thousands of families, and the supervision depends on you, me, and him”.). This slogan also makes easily recognizable arable land protection-related policies and choice architecture of people, thereby increasing the selection chance. In addition, rural slogans are an important tool and carrier for “policy to the countryside”. Rural slogans have the characteristics of wide coverage, conformity to audience awareness, and low economic costs, which are particularly suitable for arable land protection policy dissemination in rural communities. Basic-level administrative organizations have transformed arable land protection policies and regulations into an easily understandable language to facilitate comprehension and acceptance of the broad masses of farmers.

### 4.2. Heritage Motives Nudge Arable Land Protection Behavior

Stimulating home and country sentiments solves the short-sighted problem of people. However, the long period and excessively far away return on investment from future generations is also another psychological obstacle that affects the arable land protection behavior. People focus more on the current self-interest than on the future social interests (future generations), thus generally showing low willingness to protect arable land. Heritage motives theory is an economic concept that studies intergenerational exchange and wealth transfer within a family. The wealth accumulation of the micro family is through family savings, consumption, and asset allocation decisions, macro investment, and public policy choices [78]. Therefore, raising the attention of people to the interests of future generations may nudge their arable land protection behavior.

The heritage value of arable land is the allocation question of arable land resources between generations. That is to say, contemporary farmers are willing to pay a certain amount of fees to protect the arable land resources considering the arable land resources usufruct for future generations. Thus, future generations can also enjoy the effects of arable land resources. However, the main focus of agricultural policy in China has long been on the pure or narrow economic value of arable land resources. The unsustainability of arable land protection policies and the decline of arable land ecosystem service functions are easily induced due to the lack of consideration of the heritage value of arable land resources in agricultural policies. Therefore, the government should open up the useful life of arable land and provide strong guarantees for stable investments and operations of farmers by continuously extending the contracting period of arable land. In the past, capitalist landowners tended to shorten the lease term to capture the excess profits generated by the additional investment of operators in the land. Consequently, arable land operators often choose to exploit the land fertility as much as possible during the lease term. The long-term unchanging arable land contracting period guarantees the actual operating stakeholders to gain the usufruct right to the excess profits of the arable land for an extended period [79,80]. In addition, the old generation of farmers can pass on their accumulated land capital to the next generations, thus fully stimulating the enthusiasm of farmers to protect arable land. Governments at all levels can consider including elements of heritage motives when promoting the concept of arable land ecological environment to enhance awareness and behavioral level of people considering arable land protection.

### 4.3. Benefit Motives Nudge Arable Land Protection Behavior

Home and country sentiments and heritage motives aim to nudge the arable land protection behavior through the attention of people to the future of the country and future generations. In addition, rationally designing the choice architecture to realize consistent arable land protection decision-making with the interests of different stakeholders can stimulate the benefit motives of each stakeholder and then nudge the occurrence of arable land protection behavior. For example, the intensive application of chemical fertilizers and pesticides meets the need to increase food production under the guidance of production targets to a certain extent but also causes serious resource and environmental problems, such as soil compaction, soil acidification, water pollution, and excessive emission of greenhouse gases [81]. Therefore, the No. 1 Central Document in 2019 once again proposed the green agricultural development goal of achieving a negative growth in the use of chemical fertilizers. From the perspective of the efficient use of agricultural waste and the maintenance of arable land capital, organic fertilizer substitution technology, which can also promote sustainable agricultural development, is necessary to realize green agriculture [82]. However, the price of organic fertilizers on the market is generally higher than that of chemical fertilizers, and the application of organic fertilizers often requires additional capital investment. Thus, these requirements generally decrease the willingness and behavioral level of farmers to buy organic fertilizers.

In reality, farmers often simplistically divide the products on the market into “green and environmentally friendly but expensive” and “not environmentally friendly but relatively cheap”. However, this division is only the result of the excessive attention of farmers on the initial purchase cost of the product and is not the real case. Compared with traditional chemical fertilizers, many new organic fertilizers have complete nutrients and long-lasting fertilizer effects despite their high initial purchase cost. The total cost of new organic fertilizer is low due to its advantages in improving soil quality, enhancing crop quality, and reducing agricultural non-point source pollution. Life cycle cost refers to the sum of all costs related to the life cycle of the product system, including initial and operating costs [83]. If life cycle cost information can be indicated for new organic and traditional chemical fertilizers, then the preference of farmers for new organic fertilizers can be nudged, and the proportion of organic fertilizers usage can be gradually increased. Therefore, stimulating individual benefit motives can effectively resolve the “individual-society” asymmetry between the cost and benefit of arable land protection behavior.

## 5. Discussion: Controversies That May Exist in the Practice of Nudge in Arable Land Protection Behavior

The six nudge-based intervention strategies for arable land protection behavior proposed in this article reveal that nudge not only ensures the free choice of each stakeholder but also reflects the policy intentions of quantity control and quality and ecological management of arable land. Therefore, nudge is a new tool for smooth and effective policy intervention. In addition, the growth rate of fiscal revenue has slowed down while fiscal expenditure has rapidly grown after the entrance of economic development in China into the economic new normal, and cost constraints of arable land protection policy interventions have continued to increase. However, unlike traditional interventions that change arable land protection behavior of people by modifying the cost–benefit structure of decision-making at a considerable economic cost, the nudging strategies trigger only the intuition, feelings, and automatic decision-making process of individuals. Nudge can also achieve the goal of arable land protection through simple clues and small changes in the selection environment. Moreover, nudge is a low-cost intervention that can be widely used. The advantages of nudge have achieved convincing results in different countries and research fields and have shown remarkable application value. However, questions and disputes regarding the validity, reliability, and ethics of nudge frequently arise due to its novelty [84,85]. The following disputes may emerge upon the implementation of the nudging strategies of arable land protection behavior.

### 5.1. Nudge May Be Evil?

The most direct objection to nudge theory comes from “evil nudge”, which is a certain form of despotism or control threat theory. That is, people with ulterior motives use cognitive biases and other psychological laws to guide the behavior of decision makers to realize beneficial resulting behaviors to specific interest groups. For example, some local governments are accustomed to adopting “transactional”, “bribery”, and “buying-out” methods to increase the non-agricultural income of farmers. Therefore, the rights-safeguarding mechanism is not sound when its channel is rough. Moreover, farmers will feel pessimistic regarding rights protection and are inclined to assist the local government in implementing CLPP flexibly when the cost of rights-safeguarding is relatively high. However, as a method and technology, nudge has no moral and ethical issues. Any method can be abused, but such abuse is not a problem of the method itself; instead, the problem mainly lies in the purpose of the person using such a method. Restraining possible abusive behavior in the practice of nudging is necessary. Simultaneously, the positive effect of nudging methods on arable land protection behavior should be affirmed.

### 5.2. Nudge Leads to Childization?

The implementation of the nudging strategies compensates for the defects of thinking systems of people and relies on system 1 for decision-making to guide them and produce choices close to the goal of arable land protection. However, this guidance method does not transfer knowledge to the stakeholders and does not improve their decision-making skills, which hinders the accumulation of knowledge of arable land protection and the ability of independent selection of the stakeholders to a certain extent. This kind of operation will cause people to become dependent and threaten them to think naively. People who have the above doubts often overestimate the role of autonomy. In the absence of nudges, local governments also have strong political and economic demands, and their consideration focuses on developing the local economy, achieving rapid growth in fiscal revenue, and maximizing political performance during the term of office. Driven by comparative interests, an increasing number of rural laborers choose to abandon traditional agriculture and enter non-agricultural industries, and the proportion of non-agricultural income is gradually increasing [86]. Therefore, under the condition of cost–benefit, a considerable amount of critical reflection that people invest in the process of autonomous formation and adjustment does not necessarily guide individuals to make the most correct response. Thus, verifying whether nudge will lead to childization will take a long time.

### 5.3. Can Nudge Be Effective in the Long Term?

Simultaneously, some scholars argue that the role of nudging strategies has been exaggerated. In a real environment, nudging strategies may not be able to solve complex social problems [87]. The various nudging strategies of arable land protection behavior in this article are still a preliminary discussion on the theoretical level despite their capability to provide strong evidence for their effectiveness. These measures require further experiments and research by the government to evaluate their feasibility, cost, and effectiveness. Subsequent research should also further integrate the institutional design and decision-making mechanism of arable land protection policies in China, observe the cognitive behavior patterns of farmers in different regions, explain the differences in the nudging strategies formulated by local governments at different levels and regions, and then propose practical and feasible nudging strategies.

## 6. Policy Implications: Effective Use of Nudge to Promote Arable Land Protection Behavior in China

As a country with the largest population, the top priority of China is to protect arable land for national well-being and the livelihood of people. Such a priority is not only essential to achieving sustainable social and economic developments in China but is also of considerable strategic significance for ensuring world food security and stabilizing international food prices. As a rational method of behavioral science that attempts to change the psychology and behavior of people to promote social development, nudge will play a unique and irreplaceable role in this process. Considering national conditions in China when the nudging strategies are implemented is necessary to achieve the intervention goal.

### 6.1. Selection of Nudging Strategies Based on the Subdivision of Farmer Groups

Similar to traditional intervention strategies, the choice of nudging strategies should be based on the subdivision of farmer groups. Facing farmers with different characteristics, the same nudging strategies may have different effects for various intervention contents. Therefore, the policy makers of the nudging strategies should first clarify the characteristics of the target farmers. The existing literature classifies the types of farmers in a variety of ways: the types of farmers based on their employment and economic status, their decision-making behavior goals, and age as an intergenerational difference [88,89]. Under the internal and external stimuli of four new modernizations and the reform of the rural economic system in China, farmers have rapidly differentiated and transformed along various paths and methods. Significant differences are also found in the willingness and behavioral response of different types of farmers to arable land protection. For example, Xie et al. [90] divides farmers into young farmers, middle-aged farmers and old farmers according to the intergenerational differences. In addition, Xie believes that the need for livelihood security for elderly farmers makes them more dependent on arable land. Middle-aged farmers tend to achieve a state of moderate-scale operation through arable land transfer. In addition, the scale of arable land has a significant positive impact on the occurrence of arable land protection behavior, and these farmers are more inclined to adopt environmentally friendly technologies to use and manage arable land; due to fact that young farmers have more opportunities to obtain non-agricultural income and employment opportunities, they are prone to abandoning arable land. Based on the rice planting data of 537 Chinese farmers, Cai et al. [91] analyzes how the differentiation of farmers’ livelihoods affects the pesticide use status of Chinese farmers. The results of the study found that, compared with pure farmers, part-time farmers were more inclined to reduce the use of pesticides to maintain the quality of cultivated land resources. The reason is that arable land plays an important role in social security for part-time farmers, and maintaining the quality of arable land can increase the diversity of their income. Therefore, formulating different nudging strategies according to different types of farmers, mobilizing the enthusiasm and initiative of various types of farmers in arable land protection, and focusing on their subjective functions is an important guarantee for improving the effectiveness and promoting the orderly development of arable land protection.

### 6.2. Number of Options for the Nudging Strategies Should Be Appropriate

Four to five are appropriate considering the options provided by the choice architecture [92,93,94]. Providing additional options can help cater to the needs of farmers. However, the burden of decision-making on the stakeholders of arable land protection will increase with the number of options. Therefore, the designer of the choice architecture needs to balance the two above-mentioned factors according to the characteristics of farmers in practice. For example, providing an excessive number of options at once when promoting environmentally friendly technologies or green agricultural products to elderly farmers is inappropriate. This inappropriate behavior is due to the preference of elderly farmers with poor information processing capabilities to choose from fewer options than young farmers. For example, Zhao et al. [95] conducted a random sampling survey of farmers who grow grain in Jiangsu Province, China, and analyzed the differences in farmers’ willingness to choose arable land protection technologies based on micro-data. The research results show that from the perspective of individual characteristics of farmers, the age of farmers and the health of farmers have hindering effects on arable land protection technology, which is also due to the fact that arable land protection requires a certain amount of physical strength and energy. The health status, physical strength and energy of farmers have a significant impact on arable land protection behavior. Therefore, it is impossible to require elderly farmers to understand and master a certain number of arable land protection technologies in a short period of time. Xie et al. [96] used the meta-analysis method to conduct a comprehensive analysis of 77 empirical studies to reveal the influencing factors, sources of heterogeneity and influence effects of farmers on the adoption of pro-environmental technologies. The results of the study show that the gender, age, and education level of farmers are regarded by many literatures as the decisive factors for farmers to adopt pro-environmental technologies. Therefore, age has a significant negative impact on farmers’ adoption of pro-environmental technologies, suggesting that as farmers age, they are less likely to adopt these technologies. The number of options matching different characteristics of farmers is not the same. Thus, providing four to five options can be used as a general guideline without the restriction of additional factors.

### 6.3. Clarification of the External Environment That Nudges the Arable Land Protection Behavior

Arable land protection is not only a personal behavior problem but also a social problem. This phenomenon is the result of the joint influence of internal psychological resources and the external environment. If policy makers do not consider external environmental factors and blindly rely on nudge to achieve the goal of arable land protection, then its effectiveness will be severely limited. The external environment of nudge can be divided into underutilized and unprepared environments [97]. Underutilized environments mean that the central government has introduced the most stringent arable land protection system and is fully equipped with corresponding agricultural infrastructure. However, problems regarding the pessimistic effect of arable land protection and the biased implementation of arable land protection policies remain. These problems emerge due to the imperfect psychological system of the public, often making decisions that are not conducive to the arable land environment. The use of social governance methods of nudge can effectively circumvent the cognitive and motivational limitations of people at this time and guide them to act in the direction of arable land protection.

Unprepared environments refer to locations that lack agricultural supporting infrastructure. Only the nudging strategies designed for choice architecture fail in such environments. For example, agricultural infrastructure is an important material condition and core element to support the development of agricultural modernization in China and is also a crucial investment that takes advantage of cost-saving measures, such as agricultural scale operation and technological progress [98]. However, agricultural infrastructure in China is still facing problems and challenges, such as insufficient total supply, low satisfaction rate of farmers, and failure to meet the construction needs of new forms of agricultural operations effectively. Therefore, improving the efficiency of investment and financing of agricultural infrastructure, as well as the benefits of construction and use, has become the top priority for promoting arable land protection behavior. Further improvement of arable land protection policies and the construction of agricultural supporting infrastructure will help the formation of the external environment and improve its effects. Thus, policy makers can effectively evaluate the external environment of nudges to design reasonable and effective intervention programs to protect the ecological environment of arable land.

## Data Availability

Data sharing not applicable.
